# Diagnostic and therapeutic strategies in cryptococcosis: impact on outcome

**DOI:** 10.1590/0074-02760180050

**Published:** 2018-05-07

**Authors:** Timothée Boyer Chammard, Elvis Temfack, Olivier Lortholary, Alexandre Alanio

**Affiliations:** 1 Institut Pasteur, Centre National de Référence Mycoses Invasives et Antifongiques, CNRS CMR2000, Unité de Mycologie Moléculaire, Paris, France; 2 Douala General Hospital, Internal Medicine Department, Douala, Cameroon; 3 University Paris Descartes, Necker Pasteur Centre for Infectious Diseases and Tropical Medicine, Necker Hospital, Paris, France; 4 University Paris Diderot, Sorbonne Paris City, Laboratory of Parasitology-Mycology, Saint-Louis Hospital, Paris, France

**Keywords:** Cryptococcus neoformans, cryptococcal meningo-encephalitis, cryptococcal antigen, HIV infection, advanced AIDS disease, screening

## Abstract

Cryptococcosis diagnosis has been recently improved by the use of rapid cryptococcal antigen testing with lateral flow assays, which have proved sensitive and specific. Using “test and treat” screening strategies for cryptococcal disease with these tests has been showed effective in reducing cryptococcal meningitis (CM) in HIV-infected patients. Recommended induction, consolidation, and maintenance therapeutic strategy for CM is widely unavailable and/or expensive in low and middle-income settings. New therapeutic strategies, mostly using reduced duration, have recently shown acceptable outcome or are currently tested. Diagnostic and therapeutic guidelines for cryptococcal disease in limited resources countries are undergoing a paradigmatic shift.

## DIAGNOSTIC STRATEGIES

The diagnosis of cryptococcal meningitis (CM) and more broadly, disseminated cryptococcosis, is easily made by direct examination of a pelleted biological fluid using India ink with the typical observation of rounded cells measuring about 3-10 μm in diameter surrounded a thick capsule represented as a clear halo. Sensitivity of this technique is around 86% (Kambugu et al. 2008, Boulware et al. 2014b) but can be lower in HIV-negative patients in association with a low fungal burden (Dromer et al. 2007). The definitive diagnosis relies on cultures of biological samples in Sabouraud dextrose agar incubated at 30°C (Arendrup et al. 2012). Culture might be negative in case of exposure to antifungal therapy or may need longer incubation periods up to three weeks. Another easy way to diagnose cryptococcosis is the detection of cryptococcal antigen (CrAg). CrAg is composed of polysaccharide of the cryptococcal capsule which is shed in biological fluids and can be detected as a marker of the presence of the fungus. Detection of CrAg relies usually on specific monoclonal antibodies using different methods among which are latex agglutination (LA), enzyme-linked immunosorbent assays (ELISA) and most recently a lateral flow assay (LFA). CrAg detection in serum is presumptive of active cryptococcosis, especially in HIV patients and detection in cerebrospinal fluid (CSF) is diagnostic of CM (Temstet et al. 1992).

However, cryptococcosis is a sub-acute infection. Consequently, CrAg can be detected in blood many weeks before the onset of symptoms (French et al. 2002). Serum CrAg positive patients are at 25% higher risk of developing CM within the first year of ART (Letang et al. 2015, Meya et al. 2015). CrAg screening of patients before initiation of ART associated with preemptive fluconazole treatment once CrAg is positive was associated with low number of cases of CM (Longley et al. 2016). This combined strategy is associated with a significantly reduced mortality (Mfinanga et al. 2015).

As such, systematic pre-ART CrAg screening and preemptive fluconazole is recommended for those presenting with < 100 CD4 cell counts (WHO 2017). Though promising as a strategy to decrease the incidence of cryptococcal disease, especially when associated with timely ART initiation, its implementation still lags in many countries.

*CrAg detection and diagnosis of cryptococcal disease* - Commercially available CrAg detection tests are highly sensitive and specific in serum and CSF in predicting the presence of meningitis in HIV positive patients presenting with evocative symptoms. CrAg detection in serum and CSF with symptoms suggestive of CM strongly correlates with culture- and/or India ink-confirmed CM. CSF CrAg is a rapid and reliable diagnostic test for confirmed HIV-associated CM (Temfack et al. 2018). In a recently published study, the new semi-quantitative BioSynex/ Bio-Rad CryptoPS® lateral flow assay at point of screening, clearly classified patients with or without CM (Temfack et al. 2018). Of note, serum and CSF CrAg should be preferentially considered at the time of diagnosis, as there is no clear correlation between changes in antigen titre and CSF cryptococcal culture (Brouwer et al. 2005). Persistence of detectable CrAg is frequent despite clinical improvement and complete recovery of the patient. The correlation between the evolution of CrAg levels during treatment and clinical improvement remains unclear and in case of early recurrence of symptoms, CSF CrAg should also be interpreted with caution.

*Pre-ART CrAg screening and pre-emptive therapy* - In HIV-infected patients with advanced AIDS disease, the prevalence of CrAg positivity ranged from 2-21%, with a median of 6% (IQR: 5-7) (Temfack et al., unpublished observations). CrAg-positive patients who were not offered fluconazole pre-emptive therapy are at higher risk of CM and death (Desmet et al. 1989, Jarvis et al. 2009). More so, about a third of CrAg-positive asymptomatic patients have evidence of CM (Wake et al. 2018). In CrAg positive patients who received preemptive fluconazole initiated at 800 mg/day, there was a significant decrease in the relative risk of developing CM (from 21.4-6%) and of incident mortality (Temfack et al., unpublished observations).

## THERAPEUTIC STRATEGIES

The main challenge for cryptococcosis management is for disseminated disease and meningoencephalitis, knowing that the printciple of therapy is similar for both. For both clinical presentations, recommended first-line antifungal therapy is based on a three-steps strategy including induction, consolidation and maintenance phases, that was first used in a landmark Mycoses Study Group trial (van der Horst et al. 1997). The 2010 IDSA guidelines (Perfect et al. 2010) for management of disseminated disease placed amphotericin B and flucytosine as first choices for induction phase, with amphotericin B deoxycholate (0.7-1.0 mg/kg/day) given intravenously in combination with oral flucytosine 100 mg/ kg/day (at least two weeks for HIV-infected patients and organ transplant recipients, and four to six weeks for all other patients). When available, the use of liposomal formulation of amphotericin B is preferred, as it has been showed to induce less toxicity. The preferred therapy for pulmonary-limited disease, or non-disseminated disease is based on fluconazole therapy alone.

The aim of CM therapy is to improve survival thanks to rapid yeast clearance from the CSF. Quantitative clearance from CSF, called “early fungicidal activity” (EFA), is measured as the rate of yeast clearance per millilitre of CSF per day based on CFU counting. In a combined cohort of 262 patients, a slow rate of cryptococcal clearance was shown to be independently associated with increased mortality at two weeks and at 10 weeks (Bicanic et al. 2009b). Though, in a recently published meta-analysis, combining 2854 HIV-infected or non HIV-infected patients in 27 randomised CM treatment trials, the authors concluded that all-cause mortality remains the essential primary outcome to be used to assess therapy efficacy in clinical trials (Montezuma-Rusca et al. 2016).

Thereafter, we summarise the main studies and clinical trials which have helped to improve cryptococcosis outcome and survival in the last decades.

## COMBINATION ANTIFUNGAL THERAPY

*Amphotericin B with flucytosine* - The use of flucytosine combined with amphotericin B for CM therapy was already observed in a study in 1979, before the HIV pandemic, where authors concluded that the combination therapy allowed more rapid sterilisation of the cerebrospinal fluid (p < 0.001) and less nephrotoxicity (p < 0.05) than did amphotericin B alone, although results were obtained at different dosages than those currently recommended (Bennett et al. 1979).

In 1997, the Mycoses Study Group randomised, double-blind, placebo-controlled, trial involving 408 HIV-infected patients evidenced that the use of a combination therapy with amphotericin B plus flucytosine for induction therapy was associated with an increased rate of CSF sterilisation and a better outcome than amphotericin B alone, in HIV-associated CM, although without improved survival (van der Horst et al. 1997).

Aiming to confirm these results, a randomised trial conducted in Thailand, also found that the efficacy of therapy (as measured by EFA) was shown to be significantly faster in the arm in which patients were treated with the combination of amphotericin B plus flucytosine than in any of the three other arms (amphotericin B alone, amphotericin B plus fluconazole, or a triple therapy with amphotericin B, fluconazole and flucytosine), in 64 HIV-infected patients with CM (Brouwer et al. 2004).

This strategy was then also shown to be superior in the French prospective CryptoA/D study in HIV-positive or HIV-negative patients with cryptococcal infection with a mycological failure at week two of 26% in the amphotericin B and flucytosine group compared to a treatment failure of 56% with any other treatments (p < 0.001) (Dromer et al. 2007, Dromer et al. 2008).

Moreover, a three-arm, open-label, randomised trial including about 300 HIV-infected patients enrolled, demonstrated a survival benefit with this combination compared to amphotericin alone, with a reduction of about 40% in the relative risk of death at 10 weeks with addition of flucytosine (Day et al. 2013).

Finally, the recently completed “Advancing Cryptococcal Meningitis Treatment for Africa” (ACTA) trial, in which 721 HIV-infected patients with first episode of CM were randomised over nine sites in four different countries in Sub-Saharan Africa, definitively evidenced that the combination of amphotericin B and flucytosine was significantly superior to Amphotericin B and fluconazole, leading to a substantial mortality reduction [hazard ratio for death at 10 weeks with flucytosine vs. fluconazole, 0.62; 95% confidence interval (CI), 0.45 to 0.84; p = 0.002] (Molloy et al. 2018).

*Amphotericin B with fluconazole* - When flucytosine is unavailable, the combination of amphotericin B with fluconazole is still recommended (Perfect et al. 2010). In an open-label, three-arm, phase II trial including 143 HIV-infected patients, combination of amphotericin B with fluconazole 800 mg/day was found to have significantly better long-term outcomes than amphotericin B and fluconazole 400 mg/day or amphotericin alone (Pappas et al. 2009). However, this have not been observed in the study conducted by Day at al. (2013), with no statistically survival benefit found for patients receiving amphotericin with fluconazole 800 mg/day compared to those with amphotericin B alone. More so, in the ACTA trial, the treatment arm combining Amphotericin B and fluconazole had the worse outcome, clearly demonstrating that the combination of Amphotericin B and fluconazole is a not recommended for induction treatment (Molloy et al. 2018).

*Amphotericin B with voriconazole* - The use of voriconazole at the dosage of 300 mg twice daily, instead of highdose fluconazole (800 mg or 1200 mg/day) in induction therapy associated with amphotericin B was tested in a randomised 4-arms trial and found to have similar EFA, and could therefore be a theoretical option for therapy, despite its higher cost and potential interactions with other drugs including rifampicin (Loyse et al. 2012). As voriconazole is not available in most of the countries endemic for cryptococcosis, this combination would be marginally used.

*Short courses therapy* - The amphotericin B and flucytosine combination regimen remains unavailable in most parts of the world harbouring the highest burden of disease (Africa and Asia), furthermore several days of amphotericin B deoxycholate is well known to cause important adverse events, such as anaemia, renal impairment, hypokalaemia, hypomagnesemia, or phlebitis, and its administration requires inpatient hospitalisation for intravenous administration and electrolytes monitoring (Bicanic et al. 2015).

Researcher in lower middle-income settings are now working to improve induction therapy phase with combination therapy, using oral combination or reducing duration of intravenous drugs, to reduce incidence of adverse events, and try to improve survival.

In a study in Uganda, based on 30 HIV-infected patients, a short 5-day course of amphotericin with highdose fluconazole 1200 mg/day was found to have a better EFA than findings with fluconazole alone in previous studies, suggesting that shorter courses of amphotericin may be used (Muzoora et al. 2012).

The multi-centre ACTA trial, testing short courses amphotericin B deoxycholate based therapies with either flucytosine or fluconazole, also showed a better survival with a 7-day course of amphotericin B and flucytosine compared with 14-day course (HR 0.56 (0.35-0.91)) (Molloy et al. 2018). This prompted WHO experts to now recommend a 7-days amphotericin B IV therapy with flucytosine as first line induction therapy (followed by seven days of high-dose fluconazole) in low and middle-income countries (WHO 2018).

*Fluconazole and flucytosine* - Before the highly active antiretroviral therapy era, an open-label, single-arm, prospective trial studied the fluconazole (400 mg daily) and flucytosine (150 mg/kg daily) combination therapy for a 10-week antifungal therapy in 32 patients with AIDS, and found a clinical survival at 10 weeks which appeared to be better than previously reported with amphotericin B alone or fluconazole alone (Larsen et al. 1994).

More recently, a randomised clinical trial compared combination therapy with high dose fluconazole (1200 mg daily) and flucytosine (100 mg/kg daily) to high dose fluconazole alone for the 2-week induction therapy, in 41 HIV-infected patients with CM in Malawi, and found better EFA and survival for the combination therapy arm (Nussbaum et al. 2010).

Finally, the ACTA trial including 721 randomised HIV-infected patients also showed that a combination oral therapy with high dose fluconazole (1200 mg/day) and flucytosine (100 mg/kg/day) for the 2-week induction therapy was non-inferior to the standard 2-week courses of amphotericin B based therapy with flucytosine or fluconazole (Molloy et al. 2018). That made authors conclude that this regimen is an acceptable and effective option for CM therapy in Africa if amphotericin B is not available or not recommended.

## FLUCONAZOLE MONOTHERAPY

Both 2010 IDSA and 2011 WHO guidelines recommend high-dose fluconazole monotherapy (1200 mg/ day) for 10-12 weeks if amphotericin and flucytosine are not available (Perfect et al. 2010, WHO 2011).

In a cohort study carried out in Malawi using high dose fluconazole (1200 mg) as induction therapy (Gaskell et al. 2014), mortality was unacceptably high, as it was in a study using 800 mg (Rothe et al. 2013), showing that fluconazole monotherapy is not suitable as induction therapy.

A recently published CrAg screening study in Ethiopia in HIV-infected patients, highlighted that the use of high dose fluconazole monotherapy for induction phase is inadequate, leading to a 68% mortality rate at three months, in patients with CM (Beyene et al. 2017).

## USE OF LIPOSOMAL AMPHOTERICIN B

At the beginning of highly active antiretroviral therapy era, in 1997, a randomised trial comparing liposomal amphotericin B (L-AmB) and conventional deoxycholate amphotericin B in 28 HIV-infected patients with CM, observed that a 3-week course of 4 mg/kg L-AmB had a significantly earlier CSF culture conversion than a 3-week course standard amphotericin B, with similar clinical outcome and that L-AmB had significantly less renal adverse events (Leenders et al. 1997).

In a larger multi-centre trial, in which 267 patients were randomised, liposomal amphotericin B - therapy was similar in term of efficacy and overall mortality at 10 weeks, compared to conventional amphotericin B deoxycholate for HIV-associated CM. L-AmB at a dosage of 3 mg/kg/day was associated with significantly fewer adverse effects than at a dosage of 6 mg/kg/day, and compared to conventional amphotericin B (Hamill et al. 2010).

More recently, 80 participants were enrolled in the AMBITION preliminary phase II trial comparing different short courses of L-AmB with high dose fluconazole. The single dose arm was non-inferior to the standard 14-days arm in term of early fungicidal activity, compared to two-doses or three-doses arms (Jarvis et al. 2017).

Based on these results, the AMBITION phase III (ISRCTN 72509687) ongoing trial is currently taking place to evaluate whether a single high dose (10 mg/kg) of LAmB given with high dose fluconazole and flucytosine is as effective as the newly recommended (based on ACTA trial) one-week daily-dosed amphotericin B deoxycholate based induction therapy with flucytosine as an induction therapy (Molefi et al. 2015). This trial will be the largest therapeutic trial on CM with 850 patients to be enrolled over a 3-year period in five different African countries.

## ADJUVANT THERAPIES

*Steroids* - Adjuvant glucocorticoid therapy is successfully used in patients with non-fungal meningitis such as bacterial or tuberculous meningitis, and reduces mortality in some sub-groups. In CM IDSA guidelines (Perfect et al. 2010), experts recommend to consider the use of dexamethasone at high doses for patients having immune reconstitution inflammatory syndrome with severe central nervous system signs and symptoms, using a two-six-week course.

The CRYPTODEX double-blind, randomised, placebo-controlled trial, conducted in 6 countries over Africa and Asia, aimed to demonstrate the efficacy of adjunctive dexamethasone but was stopped after the enrolment of 451 patients (on an overall of 880 planned) for safety reasons (Beardsley et al. 2016). All patients received the locally-available and standard-of-care combination antifungal therapy with amphotericin B and fluconazole, and were randomised to receive either dexamethasone (intravenous for the first two weeks, and then orally, tapered doses until the 6th week) or placebo for six weeks. The primary outcome was survival until 10 weeks after randomisation. There was no difference between groups – 47% in the dexamethasone group and 41% in the placebo group died. EFA was measured and during the first two weeks of treatment, dexamethasone was significantly associated with slower rates of decline in the EFA in CSF than was placebo. Even if dexamethasone was significantly associated with a better reduction in CSF opening pressure during the first two weeks than was placebo, it was also associated with more adverse events and disability.

*Interferon-gamma* - In a prospective trial, with 90 HIV-infected patients randomised in three different arms [standard therapy with amphotericin B plus flucytosine, standard therapy with two doses of interferon-gamma (INF-γ), and standard therapy with six doses of INF-γ], an increased rate of clearance with two doses of adjunctive INF-γ than with standard therapy was shown, but there was no significant difference in mortality between groups (Jarvis et al. 2012).

*Sertraline* - Previous studies have suggested that sertraline, a selective serotonin reuptake inhibitor (SSRI), frequently used as an anti-depressant, has a real in vitro and in vivo fungicidal activity against *Cryptococcus neoformans,* as its action has a synergistic effect with fluconazole in reducing the fungal burden in brain, kidney, and spleen (Zhai et al. 2012). The first clinical open-label and dose-finding study was conducted in Uganda, recruiting 172 patients with HIV-associated CM, to assess the efficacy of adjunctive sertraline, with standard amphotericin B and high-dose fluconazole antifungal therapies (Rhein et al. 2016). Authors concluded that patients had faster cryptococcal CSF clearance and a lower incidence of immune reconstitution inflammatory syndrome and relapse than that reported in the past.

Following these results, the “Adjunctive Sertraline for the Treatment of HIV-associated Cryptococcal Meningitis (ASTRO-CM)” phase III randomised placebo-controlled clinical trial had been evaluating if sertraline dosed initially at 400 mg/day for two weeks, followed by 200 mg/day for an additional 10 weeks prior to tapering, could have an 18-week survival benefit, compared with placebo when receiving standard available induction therapy of amphotericin B deoxycholate and fluconazole 800 mg/day. The trial was stopped after enrolling 460 of the 550 patients planned for futility, showing a 18-week mortality of 52% in sertraline group and 46% in placebo group (p = 0.15) (Rhein et al. 2018).

*Tamoxifen* - Tamoxifen, the oestrogen receptor antagonist drug usually used for breast cancer, has been shown to be fungicidal and synergistic with fluconazole and amphotericin B in vitro and in vivo in a mouse model of disseminated cryptococcosis (Butts et al. 2014). At acceptably concentrations for humans, tamoxifen combined with fluconazole decreased brain fungal burden, and has demonstrated to inhibit the growth of *C. neoformans* within macrophages, which are not accessible by classical antifungal drugs. It has also the benefit to have a good oral bioavailability.

Therefore, an open-label, phase II, randomised trial (“A randomised trial of tamoxifen combined with amphotericin B and fluconazole for cryptococcal meningitis”, NCT03112031) will enrol 50 patients in Vietnam, comparing EFA according to the strategy between (amphotericin B plus fluconazole ± tamoxifen during the first two weeks).

## ELEVATED OPENING CEREBROSPINAL FLUID PRESSURE

Elevated intracranial pressure (CSF pressure > 25 cm H2O) is a usual complication of CM, secondary to a failure of CSF resorption because of obstruction by crypto-coccal polysaccharide capsule and yeasts.

In several large randomised trials (van der Horst et al. 1997, Brouwer et al. 2004, Bicanic et al. 2007) of amphotericin B-based therapy for CM, the effect of baseline CSF opening pressure was evaluated (Graybill et al. 2000, Bicanic et al. 2009a) and led to recommend to relieve by large-volume CSF drainage, performing daily therapeutic LPs if the CSF pressure is ≥ 25 cm of CSF with symptoms of increased intracranial pressure during induction therapy.

Another randomised trial comparing acetazolamide or placebo, with amphotericin B alone was prematurely stopped after recruitment of 24 patients in Thailand with CM, presenting with headache and an elevated opening cerebrospinal fluid pressure ≥ 20 cm H20, after having observed that patients who received acetazolamide developed significantly lower venous bicarbonate levels and higher chloride levels and had more frequent serious adverse events than patients who received placebo (Newton et al. 2002).

In the COAT trial, the effect of therapeutic lumbar puncture (LP) was evaluated in HIV-infected patients treated for CM, and investigators found that therapeutic LPs were associated with a 69% relative improvement in survival, regardless of initial intracranial pressure (Boulware et al. 2014a, Rolfes et al. 2014).

## ANTIRETROVIRAL THERAPY FOR HIV-INFECTED PATIENTS

Antiretroviral therapy (ART) is the corner stone of treatment in HIV-infected patients with advanced AIDS disease. In patients with CM, it is now recommended delaying ART for four-six weeks after starting antifungal therapy (WHO 2017). This deferring strategy has indeed shown to significantly improve survival, compared to early ART initiation strategy (Boulware et al. 2014a).

## NON-HIV-INFECTED PATIENTS

In solid organ transplant recipients, cryptococcal disease has different presentation, with less disseminated disease, and more lung-limited presentation (Singh et al. 2008). This could be caused by the use of calcineurin inhibitor immunosuppressive agents - such as cyclosporine or tacrolimus. These agents are synergistic with antifungals, and their use had shown better outcomes (Kontoyiannis et al. 2008). The optimal way to manage immunosuppressive regimen is to do sequential or stepwise reduction of immunosuppressive agents, and to consider decreasing corticosteroid therapy first (Perfect et al. 2010). For disseminated presentations, lipid formulations of amphotericin B are preferred to standard amphotericin B deoxycholate based therapy, given the potential risk of renal deterioration and drug interactions in these patients.

## IN CONCLUSION

Cryptococcal disease is a death-related disease in immunosuppressed patients, especially in advanced AIDS disease patients. Over the past 15 years, many studies and clinical trials have led to improve prevention, diagnosis, therapy and outcome of patients with cryptococcal meningitis. Efforts are now needed to implement those strategies in middle and low-income settings using affordable and efficient diagnostic strategies and drug regimen. These new strategies are being evaluated and will be implemented progressively to improve patient management.
